# Evolution of drought resistance strategies following the introduction of white clover (*Trifolium repens* L.)

**DOI:** 10.1093/aob/mcaf037

**Published:** 2025-03-06

**Authors:** Brandon T Hendrickson, Caitlyn Stamps, Courtney M Patterson, Hunter Strickland, Michael Foster, Lucas J Albano, Audrey Y Kim, Paul Y Kim, Nicholas J Kooyers

**Affiliations:** Department of Biology, University of Louisiana, Lafayette, 410 E. St. Mary Blvd., Lafayette, LA 70503, USA; Department of Biology, University of Louisiana, Lafayette, 410 E. St. Mary Blvd., Lafayette, LA 70503, USA; Department of Biology, University of Louisiana, Lafayette, 410 E. St. Mary Blvd., Lafayette, LA 70503, USA; School of Biological Science, Louisiana Technical University, 1 Adams Blvd., Ruston, LA 71272, USA; School of Biological Science, Louisiana Technical University, 1 Adams Blvd., Ruston, LA 71272, USA; Department of Ecology and Evolutionary Biology, University of Toronto, 25 Willcocks St., Toronto, ON M5S3B2, Canada and; Department of Biology, Grambling State University, 403 Main St., Grambling, LA 71245, USA; Department of Biology, Grambling State University, 403 Main St., Grambling, LA 71245, USA; Department of Biology, Grambling State University, 403 Main St., Grambling, LA 71245, USA

**Keywords:** dehydration avoidance, dehydration tolerance, RNAseq, invasive species, ecophysiology, adaptation, phenotypic plasticity

## Abstract

**Background and Aims:**

Success during colonization is likely to depend on growing quickly and tolerating novel and stressful environmental conditions. However, rapid growth, stress avoidance and stress tolerance are generally considered divergent physiological strategies.

**Methods:**

We evaluated how white clover (*Trifolium repens*) has evolved to a divergent water regime following introduction to North America. We conducted RNA sequencing within a dry-down experiment using accessions from low- and high-latitude populations from native and introduced ranges and assessed variation in dehydration avoidance (ability to avoid wilting) and dehydration tolerance (ability to survive wilting).

**Key Results:**

Introduced populations were better at avoiding dehydration, but poorer at tolerating dehydration than native populations. There was a strong negative correlation between avoidance and tolerance traits, and expression of most drought-associated genes exhibited similar trade-offs. Candidate genes with expression strongly associated with dehydration avoidance were linked to stress signalling, closing stomata and producing osmoprotectants. However, genes with expression linked to dehydration tolerance were associated with avoiding excessive production of reactive oxygen species and toxic bioproducts of stress responses. Several candidate genes showed differential expression patterns between native and introduced ranges and could underlie differences in drought resistance syndromes between ranges.

**Conclusions:**

These results suggest that there has been strong selection following introduction for dehydration avoidance at the cost of surviving dehydration. More broadly, trade-offs between dehydration avoidance and tolerance responses are likely to exist at both the genetic and phenotypic scales that will influence evolutionary responses and potentially limit the global spectrum of plant form and function.

## INTRODUCTION

Species introductions are occurring at a drastically increasing rate due to globalization and human commerce (Seebens *et al.*, 2017; Bonnamour *et al.*, 2021). Determining how and why some introduced species become invasive is a persistent goal for biologists that has had relatively limited success (Kolar and Lodge, 2001; Pyšek and Richardson, 2007). Introduced species almost certainly encounter some novel abiotic or biotic environmental conditions during initial colonization and must both establish and spread to become invasive despite this likely change in environment. Although historically neglected as an important evolutionary force during invasion, selection is commonly observed during successful invasions, especially for human commensal species that are introduced repeatedly and/or have high genetic variation among colonists (Huey, 2000; Maron *et al.*, 2004; Colautti and Lau, 2015; Battlay *et al.*, 2024).

Selection is key for many of the hypotheses that describe why some species become successful invaders. Stress resistance and phenotypic plasticity have frequently been hypothesized as key attributes for successful invasive species (Richards *et al.*, 2006; Gewing *et al.*, 2019). Individuals that are able to avoid or tolerate stress can better establish and spread in novel environments. Central to this hypothesis is that selection acts to remove less stress-resistant individuals from the populations. Such selection could also occur only in certain areas of the introduced range, where selection pressures are strongest. Alternatively, hypotheses such as the evolution of increased competitive ability (EICA; Blossey and Nötzold, 1995) suggest that introduced plants are removed from key selective pressures in the native range following colonization and that the strongest selection pressure is simply for rapid growth rate and high fecundity to outcompete native species.

One of the most important selection pressures for plants is water availability and associated drought events (Siepielski *et al.*, 2017). Three different non-mutually exclusive drought resistance strategies are hypothesized to evolve in environments with low or inconsistent water availability: drought escape, dehydration avoidance and dehydration tolerance (Ludlow, 1989; Kooyers, 2015; Volaire, 2018). Plants that have evolved to escape drought grow rapidly, reproduce quickly and finish their life cycles prior to or immediately following drought stress (e.g. Geber and Dawson, 1990; McKay *et al.*, 2003). Plants that are dehydration avoiders limit water loss to maintain normal functioning during drought stress. Although dehydration avoidance can be a constitutive strategy, whereby certain individuals might have high succulence, more trichomes, higher water-use efficiency or lower stomatal density (Kooyers *et al.*, 2015), avoidance can also manifest as a response to reduced water availability by lowering stomatal conductance, raising water-use efficiency and producing osmoprotectants (Geber and Dawson, 1990; Dudley, 1996). Plants with dehydration-tolerant strategies survive during periods of drought in order to reproduce following drought. These plants limit water loss during drought conditions by reducing vegetative growth, allocating resources to root growth and producing antioxidants to limit cellular damage from desiccation (Ilyas *et al.*, 2021). These responses allow plants to survive and recover following drought.

A common experimental design to tease apart variation in drought strategies between populations and/or species is the dry-down experiment (e.g. Des Marais *et al.*, 2014; Bouzid *et al.*, 2019; FitzPatrick *et al.*, 2023). In these experiments, variation in time to first flower can be considered a drought escape metric, with accessions that exhibit accelerated flowering regarded as better drought escapers (FitzPatrick *et al.*, 2023). Plants that can avoid wilting until relatively lower soil water contents can be considered better drought avoiders (Bouzid *et al.*, 2019). Plants that are capable of surviving and recovering after an extended period of wilting have greater dehydration tolerance (Bouzid *et al.*, 2019).

Although drought strategies are not mutually exclusive, negative correlations between some strategies are expected owing to physiological constraints. For instance, the rapid growth expected for drought escape requires opening of stomata in order to increase photosynthetic rate (Geber and Dawson, 1990; Dudley, 1996). However, open stomata increase transpiration and lower water-use efficiency, effectively neutralizing any dehydration avoidance strategy. Yet, the evidence for this trade-off is equivocal and seemingly context dependent (McKay *et al.*, 2003; Kooyers *et al.*, 2015). Correlations between dehydration avoidance and tolerance strategies are less explored and are often viewed as compatible responses (Volaire, 2018). However, resource allocation trade-offs could be hypothesized, because the functions necessary for maintaining water balance in a limited water environment (dehydration avoidance) are not the same resources necessary for surviving and recovering from dehydration (dehydration tolerance).

Although the molecular signals underlying the perception and integration of water stress are becoming better resolved, variation in drought-related responses is rarely understood at the molecular level (VanWallendael *et al.*, 2019). For instance, it is largely unknown whether variation in drought resistance arises through differences in sensitivity or perception of drought, integration of signals, or re-wiring of connections within gene-regulatory networks. Molecular responses to low water availability centre around the activation of the abscisic acid (ABA)-dependent and ABA-independent signal transduction pathways in response to disruption in water potential relative to the surrounding soil and/or atmosphere (Raghavendra *et al.*, 2010; Dar *et al.*, 2017; Aslam *et al.*, 2022). Increases in ABA drive a signal cascade leading to guard cell regulation and stomatal closure (Hsu *et al.*, 2021). Notably, production of ABA also induces gene regulatory cascades that lead to increases in antioxidants and osmoprotectants, such as proline, trehalose and raffinose family oligosaccharides (Strizhov *et al.*, 1997; Lunn *et al.*, 2014). The interactions of ABA with other plant hormones (specifically, antagonistic interactions with gibberellic acid and ethylene) lead to cessation of shoot growth and/or increased root growth (Wilkinson and Davies, 2010). Not surprisingly, this complex web of gene regulatory cascades has substantial overlap; for instance, products of the SNF1-related kinases 2 (*SnRK2*) gene family integrate growth rate changes, promote stomatal closure and increase osmoprotectants (Yoshida *et al.*, 2015; Belda-Palazón *et al.*, 2020). A key goal for the plant science community is to determine how these networks are manipulated to create the diversity of responses to water limitation observed within and among species (Kesari *et al.*, 2012; Des Marais *et al.*, 2014). Invasive species with divergent responses to water limitation between introduced and native areas can provide key natural experiments for examining the evolution of drought resistance.

White clover (*Trifolium repens*) is a perennial plant that is native to Eurasia but has been distributed across the world as a cover crop and forage legume during the past 400 years (Kjærgaard, 2003). Despite relatively recent introductions, there is little decline in genetic diversity in the introduced ranges of white clover (Wu *et al.*, 2021) and extensive evidence for selection and adaptation following introduction (Kooyers and Olsen, 2012, 2013; Wright *et al.*, 2018, 2021; Santangelo *et al.*, 2022; Albano *et al.*, 2024; Battlay *et al.*, 2024). Although white clover is most often associated with temperate habitats, there is extensive variation in aridity across both the native and introduced ranges, and water availability seems to be a key selective pressure (Foulds, 1977; Kooyers *et al.*, 2014; Albano and Johnson, 2023). Escape, avoidance and tolerance responses have been noted by different research groups. Drought escape via more annualized life history is favoured in lower-latitude populations in North America (Wright *et al.*, 2021). Dehydration avoidance might also be favoured in these populations, because clines in cyanogenic glucosides, potentially key sources of nitrogen during drought conditions, have evolved following introduction to several different continents. In each introduced region, the proportion of plants possessing cyanogenic glucosides increases in more arid areas (Kooyers and Olsen, 2013, 2014). Finally, agricultural breeders frequently target persistence following drought as a key trait for selection in agricultural lines (Marshall *et al.*, 2001; Jahufer *et al.*, 2002). However, differences in drought resistance strategies have not been examined systematically across native and introduced populations.

Here, we use a manipulative dry-down experiment to examine how populations across the native and introduced ranges of white clover resist drought. Specifically, we examine the relative degree of dehydration avoidance and tolerance among populations from low- and high-latitude populations in the native and introduced range using volumetric water content (VWC) at wilting as a measure of dehydration avoidance and ability to recover following wilting as a measure of dehydration tolerance. We also examine differential expression between well-watered and dry-down plants at a time point when the dry-down lines begin to experience drought stress. We use these data to address four questions. First, do populations from introduced areas or from more arid areas have more substantial drought resistance strategies? The stress tolerance hypothesis suggests that introduced plants should be better stress tolerators, but EICA might suggest the opposite. Second, are there trade-offs between dehydration avoidance and tolerance strategies such that plants that are good dehydration avoiders are also poor dehydration tolerators? Third, do native and introduced populations exhibit different patterns of gene expression in response to early drought stress? That is, differentially expressed genes between well-watered and drought treatments may or may not vary among ranges and populations. Fourth, how do expression differences control variation in dehydration avoidance and tolerance strategies? Together, these experiments provide insight into the molecular mechanisms underlying the evolution of drought resistance across a rapid invasion.

## MATERIALS AND METHODS

**Fig. 1.**
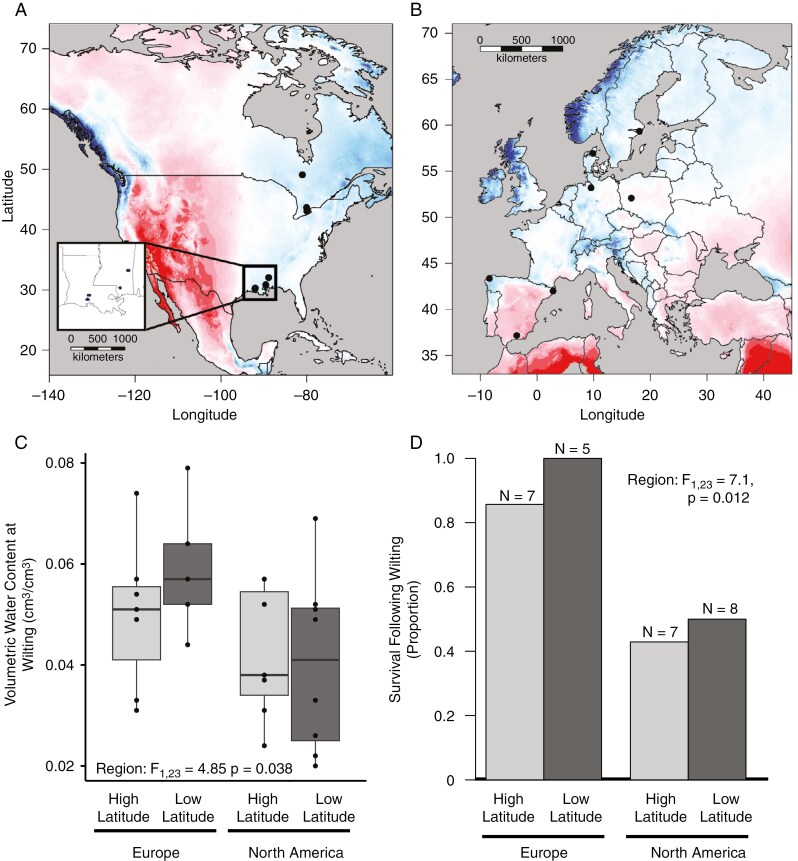
Variation in drought strategies between native and introduced populations of white clover. (A and B) Map of sampling locations in the native and introduced ranges. Black points represent sampling locations. Colour raster represents annual aridity index, with warmer colours indicating more xeric environments. Inset map depicts low-latitude North American populations. (C) Boxplots of variation in wilt VWC within low- and high-latitude populations in the native (European) and introduced (North American) regions. Each point is an individual. Box edges represent the interquartile range, the centre line in the box is the median, and the whiskers represent 1.5 times +/- than the interquartile range. (D) Barplots identify the proportion of individuals that survived wilting from each latitude–region contrast. Numbers above the bars are the number of individuals surveyed in the dry-down treatment.

We performed a manipulative experiment to examine variation among drought strategies and the transcriptomic drivers of such variation within and among the native and introduced ranges of white clover, *T. repens*. We planted seed collected from three or four populations from low latitude and high latitude in the native and invasive range (total of 14 populations; Fig. 1; Innes *et al.*, 2022). Seeds for each population had been pooled from ≥20 different maternal lines. There is extensive genetic variation within populations within each region, but very little differentiation among populations within regions (Kooyers and Olsen, 2012; Wu *et al.*, 2021; Battlay *et al.*, 2024). Thus, we treat individuals as independent samples within our analysis. For each population, we used latitude and longitude to extract WorldClim2 variables (Fick and Hijmans, 2017) and annual aridity and evapotranspiration data from CGIAR (Zomer *et al.*, 2008).

Seeds were scarified and planted into round pots (diameter, 10.16 cm; volume, 480 cm^3^) filled with PRO-MIX LP-15 soil presaturated with water. We did not inoculate soil with *Rhizobia* bacteria. Pots were placed in AR-66L2 growth chambers (Percival Scientific, Perry, IA, USA) and misted daily to promote germination. Growth chambers were set to a constant 22 °C, with a 14 h–10 h day–night cycle. Germinants were randomized within well-watered and dry-down treatments (well-watered = 26 individuals, dry-down = 25 individuals; Supplementary Data Table S1). Plants were rotated within chambers every 3 days to minimize microenvironmental variation. Prior to treatments and within the well-watered treatment (control), we saturated pots with water every 5–7 days depending on when pots began dry-down; this regime prevents water limitation without overwatering. Plants were grown for 6 weeks to accumulate above- and below-ground biomass. At 6 weeks, all pots were saturated by bottom watering. The number of leaves on each plant was counted, and one pot was excluded from further analysis because it was substantially larger than the other plants. Plants in the dry-down treatment did not receive additional water. Each day, we assessed VWC in each pot using an SMT150T soil moisture meter (Dynamax, Houston, TX, USA). Well-watered and dry-down treatments functioned as expected, with separation in VWC between treatments after control plants were resaturated on day 9 (Supplementary Data Fig. S1).

To examine dehydration avoidance, we recorded the day and the soil moisture at which each plant in the dry-down treatment wilted (hereafter, ‘wilt VWC’). We assessed wilting visually; a plant was considered wilted when all leaves exhibited turgor loss, with stems noticeably drooping. To determine dehydration tolerance, we determined the propensity to recover following wilting (hereafter, ‘wilt survival’). Forty-eight hours after wilting, we resaturated the pots of each wilted plant by bottom watering for an extended period of time (~1 h). We then resurveyed plants for survival 3 days (72 h) after resaturation. Survival was measured as a discrete variable (yes/no), with any revived green tissue or new growth counting as survival.

### Manipulative experiment statistics

All statistics were conducted in R v.4.2.2 (R Foundation for Statistical Computing, Vienna, Austria), with univariate linear models (LMs) and generalized linear models (GLMs) implemented with the lm() or glm() functions, respectively. Initially, to examine whether plant size influenced either dehydration avoidance or tolerance, we examined associations between either wilt VWC or wilt survival and the number of leaves at the beginning of the experiment. Neither wilt VWC nor survival was significantly associated with the number of leaves (Supplementary Data Fig. S2). The models below produced qualitatively similar results whether or not the number of leaves at day 1 was included as a covariate.

We examined whether variation in dehydration avoidance and tolerance was associated with range (native/introduced), latitude (low/high) or their interaction using GLMs. Specifically, we modelled wilt VWC and wilt survival as response variables within a GLM, with range, latitude and range–latitude interaction as factors. Wilt VWC was modelled with a Gaussian distribution with an identity link, and wilt survival was modelled with a binomial distribution with a logit link. Statistical significance was determined through ANOVA using a type II sum of squares implemented with the *Anova()* function (*car* library; Fox *et al.*, 2013).

Latitude across continental ranges might not be strongly related to water availability or aridity of populations and thus might not be the main factor driving variation in drought strategies. To examine whether variation in drought strategies was related to differences in precipitation or aridity, we examined associations between wilt VWC and wilt survival with annual aridity index, potential evapotranspiration and the coefficient of variation in annual precipitation (bio15). Distributions and links of models and assessment of statistical significance were the same as the above models. The number of leaves on day 1 of the experiment was included as a covariate in each analysis, because there were weak associations between each climatic variable and initial plant size (Supplementary Data Fig. S3).

To explore potential trade-offs between dehydration avoidance and tolerance, we examined associations between wilt VWC and wilt survival using a GLM (binomial distribution, logit link). Ability to recover from wilting was the response variable and VWC at wilting the predictor variable. Significance was tested with an ANOVA as above, and model fit was assessed using the *adjR2()* function (glmToolbox package; Vanegas *et al.*, 2024).

### RNA sequencing analysis

We used an RNA sequencing (RNAseq) analysis to examine differential expression underlying variation in drought strategies. During the manipulative experiment, leaf tissue from two healthy and recently unfolded leaves was flash frozen in liquid nitrogen 10 days after the dry-down treatment began from plants in both the well-watered and dry-down treatments. Tissue sampling was done during a 2 h period in the afternoon, with order of collection randomized within each treatment. Total RNA was extracted with Direct-zol RNA Miniprep (Zymo Research, Irvine, CA, USA). RNAseq libraries were constructed with QuantSeq 3′ mRNA-Seq FWD prep kit (Lexogen, Vienna, Austria) using an input of ~415 ng total RNA per sample following the manufacturers’ protocols. Two samples had low RNA concentrations, and we followed Lexogen’s modified procedure for low-input samples. Four samples from the manipulative experiment did not have RNAseq libraries constructed owing to loss of labels during shipping or near-zero read counts (RNAseq *n* = 47; Supplementary Data Table S2). All individuals were barcoded and multiplexed in a single tube. We sequenced this library on a single HiSeqX lane (PE 150 bp reads) through Novogene (Sacramento, CA, USA). A second round of multiplexing and sequencing was conducted for a subset of samples that had low data yield in the first round, and files from both rounds were merged subsequently. Raw sequenced reads per individual averaged 37 241 613 reads (s.d. 16 534 847; Supplementary Data Table S2).

Fastp v.023.4; (Chen *et al.*, 2018) was used to trim adapters and poly-A tails. Microbial RNA contamination was removed by aligning each sample to all fungal and bacterial assembly genomes within the NCBI database using bowtie2 v.2.5.1 (Langmead and Salzberg, 2012). The bacterial and fungal genome database was indexed with the 2.2.4 release of bowtie2. The remaining reads that did not align to the contamination database were presumed to be white clover RNA reads. A transcriptome was created from the *T. repens* reference genome (Santangelo *et al.*, 2023) using gffread v.0.12.7 (Pertea and Pertea, 2020). A full decoy-aware transcriptome was constructed to mitigate specious mapping of reads that occur from unannotated genomic loci that are similar to the annotated transcriptome, and a mapping-based index was then constructed using a *k*-mer hash over *k*-mers of length 31 using Salmon v.1.10.2 (Patro *et al.*, 2017). Read mapping to the transcriptome and quantification were then performed with Salmon v.1.10.2 in mapping-based mode using the *T. repens* transcriptome and RNA sequences. There was a clear issue with DNA contamination, because only 3.83 % of reads per individual aligned to the transcriptome (s.d. 1.0 %; Supplementary Data Table S2); however, this should not bias our comparative analysis (differential expression or transcript abundance–phenotype associations), which relies on read counts of each transcript.

### Quantifying expression across treatment, range and latitude

We used DESeq2 (Love *et al.*, 2014) to test for differences in transcript abundance between dry-down and well-watered treatment groups, between the native and introduced ranges, and between high- and low-latitude populations. We controlled for the VWC at the time of tissue collection by treating it as a covariate in the DESeq2 model (i.e. transcript ~ treatment × latitude × range + soil VWC). The false discovery rate (FDR) for each gene was calculated, and a transcript was then categorized as differentially expressed if the FDR was <0.1. Concordance of gene expression responses to drought between ranges and latitudes was determined by comparing the coefficient estimates of protected one-way ANOVAs. That is, we conducted ANOVAs for each gene found to be differentially expressed between the well-watered and dry-down treatments, with relative expression as the response variable and either range or latitude as the predictor variable.

All transcripts were used for weighted correlation network analysis (WGCNA) to determine gene modules and co-expression networks within the WGCNA v.1.72-5 library (Langfelder and Horvath, 2008). To achieve homoscedasticity amongst transcript abundance, the variance stabilization transformation from the fitted dispersion–mean relationships was calculated with DESeq2, which was then used to transform transcript count data by dividing by the size factor. Normalized transcript counts were then analysed with WGCNA using a soft-thresholding power of nine and assuming that biological networks follow a scale-free structure (Barabási and Albert, 1999). Modules were named by colour, as is standard for this type of analysis. The sets of genes within each resulting module were then cross-referenced with the ‘biological process’ Gene Ontology (GO) terms (Ashburner *et al.*, 2000) of *Arabidopsis thaliana* and used for gene enrichment analysis with an FDR cut-off of 0.1. Fold enrichment, −log_10_(FDR) and the number of genes for each GO term were then calculated. Genes belonging to the same module were then compared with the Kyoto Encyclopedia of Genes and Genomes (KEGG; Kanehisa and Goto, 2000) to visualize expression pathways.

### Identifying genes associated with wilt VWC and wilt survival

GLMs were constructed to quantify the correlation of transcript abundance with either wilt VWC or wilt survival. Initially, transcripts of drought treatment samples were standardized to acquire the relative expression for each gene. Univariate GLMs included relative expression as the response variable and either wilt VWC (Gaussian distribution, identity link) or wilt survival (binomial distribution, logit link) as predictor variables, with VWC at the start of the experiment as a covariate. Significance was then tested using ANOVA as above. To determine the set of phenotypically significant genes that are co-expressed, genes significantly associated with each phenotype were compared with the previously calculated WGCNA network. We also determined the location of these genes within the white clover genome (Santangelo *et al.*, 2023) via *blastn* (Altschul *et al.*, 1990).

Finally, we determined which of the genes associated with phenotype were also differentially expressed between ranges or across latitudes to identify genes likely to be involved in adaptation following introduction or in divergent climatic conditions. We conducted GLMs modelling relative expression as a function of traits and either range or latitude. Specifically, relative expression was the response variable, and trait value (wilt VWC or wilt survival), spatial group (range or latitude) and the interaction between trait and group were included as predictors. Genes with relative expression that is explained by trait–group interaction are good candidates for producing the phenotypic variation observed in nature.

## RESULTS

Drought resistance strategies varied across ranges of white clover but not across latitudes. Wilt VWC was lower in North American populations than in European populations (*F*_1,23_ = 4.8, *P* = 0.03; Fig. 1B), indicating greater drought avoidance in introduced populations. However, the effect size of this difference was low, because the difference in mean wilt VWC was only 1.3 % lower water content at wilting in the North American populations. There were no differences among low- and high-latitude populations nor any interaction between introduction and latitude impacting dehydration avoidance (Supplementary Data Table S3). Dehydration tolerance also differed among native and introduced ranges, with native regions better able to survive wilting (χ^2^ = 7.09, *P* = 0.012; Fig. 1C). All but one of the plants from the native range were able to recover following wilting (11 of 12 plants), but only 46.7 % (7 of 15) plants from the introduced North American range recovered. There were no differences among low- and high-latitude populations nor any interaction between introduction and latitude impacting dehydration tolerance (Supplementary Data Table S2).

There were strong associations between the abiotic environment of the site where each line originated and dehydration tolerance, but little correlation between abiotic variables and dehydration avoidance. Dehydration tolerance was associated with the aridity index, with plants from areas having a higher annual aridity index being more likely to survive wilting (χ^2^ = 6.2, *P* = 0.013; Supplementary Data Fig. S4). This pattern was driven by the low-latitude populations from the native range, which all recovered following wilting. These low-latitude populations, largely from Spain, had relatively high evapotranspiration, low annual precipitation and higher variation in precipitation. Wilt VWC was not significantly associated with any abiotic variables (Supplementary Data Fig. S4; Table S4).

**Fig. 2.**
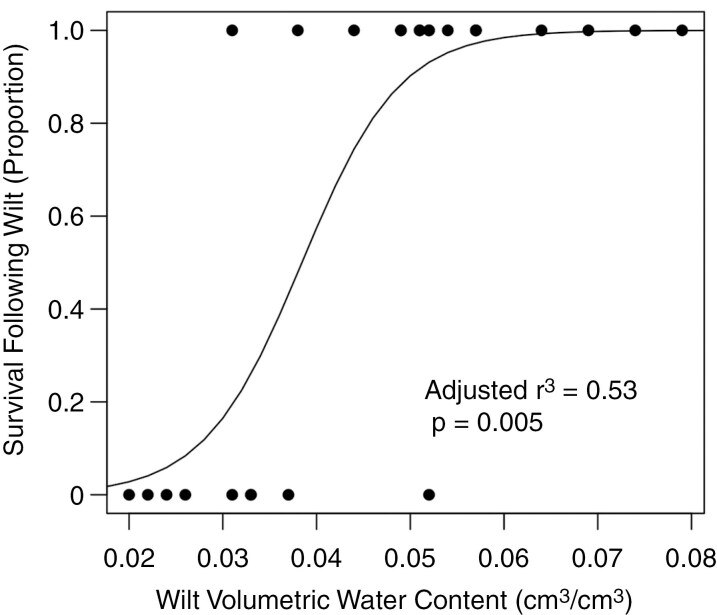
Phenotypic trade-off between dehydration avoidance and tolerance. Lower wilt VWC indicates greater dehydration avoidance, whereas higher wilt survival indicates greater dehydration tolerance.

There was a strong trade-off between dehydration avoidance and tolerance. Plants that wilted at lower VWC were less likely to recover after wilting (adjusted *r*^2^ = 0.53, *P* = 0.005; Fig. 2). All four plants that wilted at the lowest VWCs did not survive after wilting. Moreover, six of the seven plants with the lowest wilt VWC also did not survive wilting.

### Differential expression across the native and introduced ranges

Many genes were differentially expressed between dry-down and well-watered treatments, high- and low-latitude populations, and native and introduced ranges (Supplementary Data Table S5). Across predictor variables, there were 19 344 differentially expressed transcripts out of 86 723 sequenced transcripts, corresponding to 2253 of 5001 unique genes. A plurality of differentially expressed transcripts were differentially expressed within all three contrasts (treatment, range and latitude; 35.3 %; Supplementary Data Fig. S5). An additional 29.4 % of transcripts were differentially expressed in both range and latitude contrasts, but not in the treatment contrast. A far lower percentage of differentially expressed transcripts were differentially expressed in only one contrast (latitude, 13.4 %; treatment, 5.6 %; range, 10.0 %). The large union between the three contrasts in differentially expressed transcripts indicates that different drought responses are likely to be occurring across latitudinal gradients and ranges.

We examined co-expression between differentially expressed genes to determine how gene expression networks are influenced by drought treatment and vary across range and latitude.

Eight modules were discovered from WGCNA, including 5.77 % of transcripts (Table 1; Supplementary Data Table S5). Expression profiles of normalized transcript counts showed that the ‘turquoise’ module had elevated transcript counts in samples within the dry-down treatment, in contrast to all other modules, which exhibited relative uniformity across sample types (Table 1; Supplementary Data Fig. S6). Gene enrichment analysis of the turquoise module revealed marginal or significant enrichment for many GO terms typically associated with drought resistance, including hyperosmotic stress, proline metabolic process, response to oxygen-containing compounds and response to water deprivation (Supplementary Data Table S6). Within the turquoise module, 41 of the 53 genes (77.4 %) exhibited a significant latitude-by-range interaction (24 homologues exhibited significantly different range responses between high and low latitudes); substantially higher than the genes not in modules (18.2 %; Table 1). These results suggest that genes within the turquoise module might underlie adaptation to climate across the native and introduced ranges.

**Table 1.** Breakdown of the genes and module associations for each phenotype and across space

Module	Number of transcripts	Wilt VWC	Wilt survival	Latitude	Range	Latitude X Range
No module	45,916 (4360)	2066 (557)	1201 (336)	2994 (854)	1076 (309)	3895 (875)
In module	4337 (662)	346 (89)	240 (62)	603 (169)	170 (51)	592 (130)
Brown	2298 (428)	158 (50)	110 (29)	426 (134)	97 (28)	221 (54)
Red	122 (36)	8 (1)	7 (2)	20 (5)	5 (1)	15 (4)
Yellow	259 (45)	3 (1)	7 (1)	16 (5)	3 (2)	50 (9)
Grey	821 (120)	52 (6)	28 (5)	37 (8)	34 (11)	36 (6)
Turquoise	285 (53)	86 (28)	59 (15)	70 (12)	8 (0)	196 (41)
Green	138 (31)	5 (1)	5 (2)	8 (2)	8 (4)	7 (1)
Blue	324 (86)	27 (5)	21 (8)	19 (8)	15 (6)	51 (20)
Black	91 (20)	7 (0)	3 (0)	7 (1)	0 (0)	16 (5)

The number outside the parentheses refers to the number of transcripts that are associated with the phenotype or differentially expressed across latitude or range contrasts. The number within the parentheses refers to gene homologues that are associated with the phenotype or differentially expressed across latitude or range contrasts.

### Differentially expressed genes are associated with dehydration avoidance and tolerance

Expression of a large number of genes was associated with wilt VWC and wilt survival (635 and 392, respectively; 198 shared; Supplementary Data Table S7). The overlap between this group and genes that were differentially expressed within the dry-down vs. well-watered contrast represented the best candidate genes for impacting dehydration avoidance and tolerance strategies. A promising number of genes were found in this overlapping group: 47 differentially expressed genes were associated with wilt VWC and 17 differentially expressed genes with wilt survival (7 shared between phenotypes). These genes were found disproportionately in the turquoise module: 80 % of gene homologues for wilt VWC and 50 % of gene homologues for wilt survival.

**Fig. 3.**
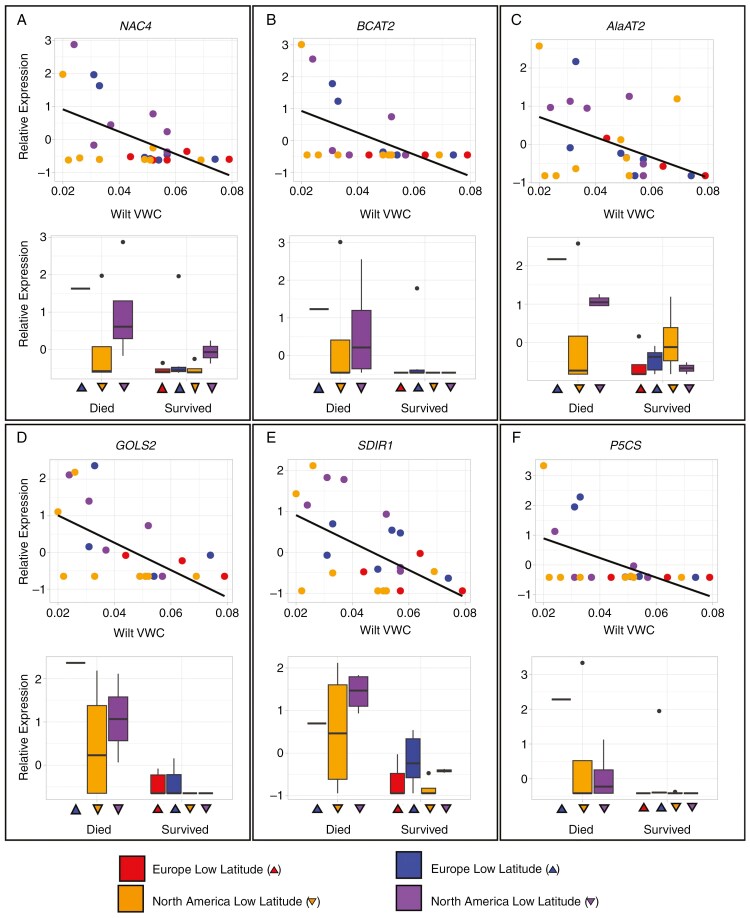
Association between candidate gene expression and dehydration avoidance or tolerance phenotypes. Lower wilt VWC indicates greater dehydration avoidance, whereas higher wilt survival indicates greater dehydration tolerance. Points and bars are coloured by latitude and range: low-latitude Europe = red, high-latitude Europe = blue, low-latitude North America = orange, high-latitude North America = purple. Each point represents a different individual. Gene Abbreviations: *AlaAT2*, alanine aminotransferase 2; *BCAT2*, branched chain amino acid transaminase 2; *GOLS2*, galactinol synthase 2; *NAC4*, nascent polypeptide-associated complex; *P5CS*, delta 1-pyrroline-5-carboxylate synthase; *SDIR1*, Salt and Drought-Induced Ring Finger1. Wilt VWC units are cm^^3^^/cm^^3^^.

**Fig. 4.**
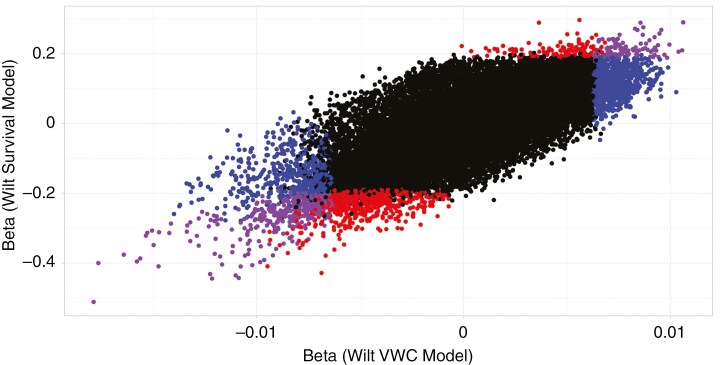
Correlations between model beta estimates from wilt VWC and wilt survival associations with RNAseq. Each point represents estimates for one transcript. Transcripts with purple points have a significant associations between expression and both phenotypes, blue points have a significant association between expression and wilt VWC, red points with wilt survival, and black points with neither phenotype.

From this initial group, we identified several strong candidate genes by examining genes with extreme effect sizes on each phenotype that are also known drought-associated genes in other species. Initially, we examined genes that were more highly expressed in plants with low wilt VWCs as genes potentially involved in dehydration avoidance. Several genes from the turquoise co-expression network had the greatest negative beta estimates for wilt VWC. These included multiple homologues of *NAC4* and *BCAT2* (Fig. 3A, B). Notably, expression of these genes was also strongly associated with death after wilting, i.e. the most negative beta estimates for dehydration tolerance. Several other strong candidate genes had this same strong trade-off between phenotypes, including homologues of *AlaAT2*, *GOLS2*, *SDIR1* and *P5SC1* (Fig. 3C–F). In fact, all transcripts that had significant associations with wilt VWC or wilt survival had either negative beta for both wilt VWC and wilt survival or positive betas for both traits (α = 0.05, 582 transcripts and 170 homologues; Fig. 4). We discuss gene functions in more depth in the Discussion, but wilt VWC-associated genes are strongly related to stress signalling via both ABA-dependent and independent pathways, stomatal responses and production of various osmoprotectants.

**Fig. 5.**
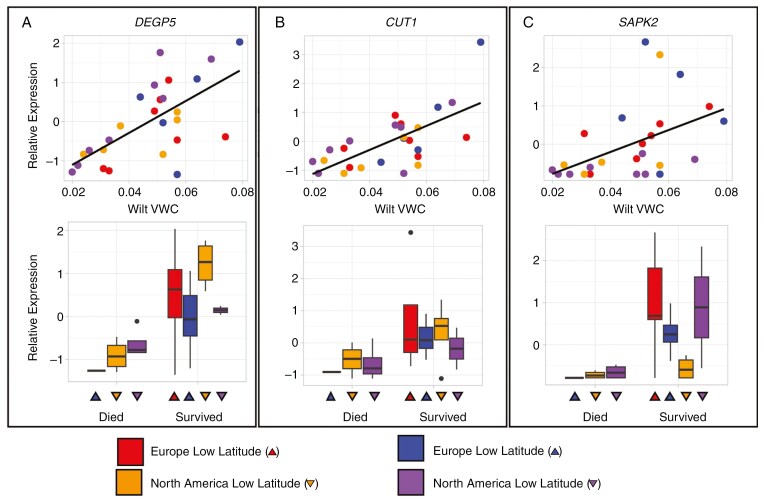
Candidate genes associated with wilt survival. Lower wilt VWC indicates greater dehydration avoidance, whereas higher wilt survival indicates greater dehydration tolerance. Points and boxplots are coloured by latitude and range: low-latitude Europe = red, high-latitude Europe = blue, low-latitude North America = orange, high-latitude North America = purple. Each point represents a different individual. Gene abbreviations: *CUT1*, Cutinase 1; *DeGP5*, degradation of periplasmic proteins 5; *SAPK2*, sucrose non-fermenting 1-related kinase 2. Wilt VWC units are cm^^3^^/cm^^3^^.

Genes with expression strongly associated with wilt survival had functions more explicitly associated with detoxifying reactive oxygen species (ROS) and negative products of photorespiration and with continued light harvesting. The gene with the strongest association with wilt survival was a homologue of *DegP5*, a gene that dampens Ca^2+^ signalling associated with stress responses and that is associated with reduced ROS (Fig. 5A). This gene also had the strongest positive association with wilt VWC of any gene (i.e. high expression of this gene was linked with wilting at a high VWC). Expression of a homologue of *CUT1* (*Cutinase 1*), a gene that impacts cuticle wax biosynthesis, was also strongly associated with greater survival following wilting. One additional candidate gene, a homologue of *SAPK2*, also had among the highest associations with increased wilt survival and higher wilt VWC (Fig. 5C). Other genes, including homologues of *PGLP2*, *CAB7* and *LHCA6*, with expression strongly associated with wilt survival, were more weakly associated with wilt VWC. These genes have less clear associations with known drought responses. Together, these candidates suggest that genes associated with lower wilt VWC include those typically associated with dehydration avoidance, whereas genes associated with wilt survival are associated with increasing photosynthesis and protecting tissues against the harmful bioproducts of stress responses.

### Expression patterns associated with phenotypic differences between ranges

Although many genes are associated with dehydration avoidance and tolerance phenotypes, a limited subset of these candidates have the potential to drive the differences in dehydration avoidance or tolerance between ranges. We examined which of the candidate genes linked to dehydration avoidance or tolerance also had differential expression across ranges or latitudes. As was the case with many genes in the turquoise network, relative expression of *BCAT2* in the drought treatment was greater in high-latitude populations, exhibited variable expression in low-latitude North American populations and had very limited expression in the low-latitude European populations (range:latitude, log_2_ fold change = 30, *P* = 4.83 × 10^−9^; Fig. 3; Supplementary Data Fig. S7). Similar patterns existed for both *P5CS* (range:latitude, log_2_ fold change = 29.9, *P* = 8.28 × 10^−11^) and *GOLS2* (range:latitude, log_2_ fold change = 18.5, *P* = 0.002). To determine other genes that might create phenotypic differences between ranges or across latitudes, we identified genes whose relative expression was best explained by an interaction between wilt VWC or wilt survival and range. Many genes (22.8 % of genes surveyed; Supplementary Data Table S8) exhibited such interactions, including 46 of the 52 genes in the turquoise network. These results suggest that the molecular variation underlying phenotypic differences between ranges either involves many loci of varying effect sizes or are large effect variants that impact hormonal stress responses and multiple gene expression networks.

## DISCUSSION

During invasions, plants encounter novel environments and may need to acclimatize or adapt to succeed. Here, we identify differences in drought resistance strategies that have evolved following the introduction of white clover to North America from its native range in Europe. North American populations have stronger dehydration avoidance responses, whereas European populations have stronger dehydration tolerance responses. These differences appear to be shaped by the abiotic conditions in each region and by a strong trade-off between dehydration avoidance and tolerance. We also identify a number of strong candidate genes underlying each of these phenotypes, with a significant proportion falling into a single co-expression network. Most of these genes have expression profiles whereby higher expression improves either dehydration avoidance or tolerance while decreasing the other phenotype. Additionally, the limited subset of genes that are associated with both a drought resistant phenotype and exhibit differential expression between native and introduced ranges represent the best candidates for identifying a molecular basis of drought adaptation following introduction. We discuss these results below in the context of invasion biology and the evolutionary genetics of adaptation to plant abiotic stresses, two fields that overlap but are rarely synthesized.

### Evolution of drought resistance during invasions

The roles of selection and adaptation during invasions are now widely documented, with many species, including white clover, rapidly evolving climate-associated clines following introduction (e.g. Kooyers and Olsen, 2012; Colautti and Barrett, 2013; Battlay *et al.*, 2023). However, generalization of which traits are favoured during invasions is less clear (Kolar and Lodge, 2001). Different trait-based hypotheses suggest that rapid growth (EICA; Blossey and Nötzold, 1995) or stress tolerance might be favoured in introduced regions (Gewing *et al.*, 2019). Our results are partly consistent with both hypotheses but are far more nuanced, because drought resistance is conferred through many different mechanisms. Introduced North American populations wilt at lower VWCs than populations from the native European range but also are less able to tolerate wilting than native populations (Fig. 1). Thus, North American populations are likely to be more dehydration avoidant than European populations. Greater dehydration avoidance might allow plants to maintain growth during periods of low water and high evapotranspiration. This would be consistent with devoting more resources to rapid growth within the introduced range, in addition to being more resistant to initial water stress. This nuance might account for some of the mixed evidence for the EICA within the literature (Rotter and Holeski, 2018).

Differentiation between native and introduced ranges in drought syndromes within a common garden is indicative of natural selection. However, differences in drought resistance strategies between ranges could also result from demographic events associated with colonization, (i.e. genetic bottlenecks; Dlugosch *et al.*, 2015) or maternal effects (Roach and Wulff, 1987). However, we view this possibility as unlikely, because white clover has high effective population sizes with limited genetic differentiation between populations worldwide (Kooyers and Olsen, 2012; Wu *et al.*, 2021; Kuo *et al.*, 2024) and has little evidence for bottlenecks occurring following introduction (Battlay *et al.*, 2024). Maternal effects are also likely to be minimal, because there was limited variation explained by population within regions, and plants were no longer young juveniles at the time of the experimental manipulation. Alternatively, our experiments might be conservative regarding our inference of natural selection, because our sample size was limited by growth chamber space. With greater statistical power, our experiment might have uncovered more nuanced differences across latitudes within regions (e.g. Europe; Fig. 1). Together, this suggests that natural selection is the most likely evolutionary force creating divergence among drought syndromes between native and introduced ranges.

We suggest that the evolution of greater stress avoidance to facilitate rapid growth might be a pattern frequently observed across invasions of perennial plants. Introduced species are likely to encounter different abiotic regimes in their new range. Invasive populations of annual plants often evolve more rapid life histories (Colautti and Barrett, 2013; Battlay *et al.*, 2023), but most perennial plants might not be able to complete a life cycle rapidly. Although some perennial plants can evolve annual life histories in stressful areas (Hall and Willis, 2006; Wright *et al.*, 2021), most perennial plants probably need to survive stresses in order to establish and reproduce. There are few plant systems where the evolution of drought avoidance or tolerance has been assessed across native and introduced regions. These examples largely consist of species that exhibit clear differentiation in drought resistance strategy between ranges. For instance, camphorweed (*Heteroteca subaxcillaris*) has drastically greater root growth in its invasive range in Israel than in its native range, North America, which is likely to be a benefit both for low water availability and for structural integrity in arid dune environments in Israel (Sternberg, 2016). Jerusalem artichokes (*Helianthus tuberous*) have evolved extreme clonality phenotypes in their introduced range (Europe) to accommodate a shift to a more aquatic environment (Bock *et al.*, 2018). Apart from plants, a greater tolerance or plasticity to stressful novel environments is often observed; for instance, anoles (*Anolis cristatellus*) from the introduced range survive colder temperatures than those from the native range (Leal and Gunderson, 2012). We suggest that invasive species should be used more widely for identifying natural phenotypic and genomic variation in drought resistance.

Although our results are consistent with classic invasion biology theory, they also make sense in the context of environmental characteristics of the populations. The populations involved in this experiment have very different growing seasons and historical climatic features (Supplementary Data Table S1). Southern European populations come from a Mediterranean climate with cool and temperate winters and hot dry summers. Relative to other locations, there is little precipitation throughout the year and almost no rainfall during summer months. A long summer with little rain is the ideal condition for the evolution of dehydration tolerance in a perennial plant that is likely to need to survive across multiple growing seasons (Kooyers, 2015). Alternatively, eastern North America has more substantial rainfall throughout the growing season, with drought stress likely to occur during shorter stretches without rain (Supplementary Data Table S1). These conditions might favour the evolution of an avoidance strategy, because additional rainfall is likely to happen (Supplementary Data Fig. S4). Consistent with these predictions, we observe the strongest dehydration avoidance strategy (i.e. wilt survival) in low-latitude European populations that have high aridity indexes and seasonal precipitation and the strongest drought avoidance strategies (i.e. wilt VWC) in North American populations that have more predictable precipitation (Supplementary Data Fig. S4). Thus, our results support that precipitation regime is a key agent of selection that might drive evolution following introduction.

Interestingly, we observe only weak differences in drought resistance strategy between high- and low-latitude populations of white clover in the native or introduced ranges. In both ranges, on average, lower-latitude populations are less dehydration avoidant but more dehydration tolerant. We suggest that the differences in precipitation regime are larger between ranges than across latitudes, particularly in North America. However, this result is surprising, because previous work documents differentiation in life-history strategy evolving across the North American latitudinal gradient, with warmer southern populations adopting a more annualized life history (Wright *et al.*, 2021; Kuo *et al.*, 2024). We note that our small sample size has the power to detect only major effects, and future larger studies might be able to parse differences in stress responses across latitudinal gradients. Additionally, our experiment was not designed to examine drought escape responses, which might contribute to the annualized life history observed in other studies.

### Molecular basis of variation in drought tolerance and avoidance

The molecular basis of drought resistance has been examined extensively in crop plants and model systems, but there has been limited success in determining the genes that underlie variation in natural populations (Des Marais *et al.*, 2013; VanWallendael *et al.*, 2019). Here, we find several candidate genes that are likely to influence dehydration avoidance and tolerance responses across both invasive and native ranges despite relatively few mapped reads from only a single tissue type (Fig. 3). Notably, the turquoise co-expression network is strongly enriched for gene ontologies related to drought responses (i.e. response to abiotic stress, response to water and proline synthesis). A high proportion of the genes within this network are also associated with wilt VWC, including some of the strongest candidate genes. Interestingly, these genes represent a diverse set of drought-related functions in other organisms, including stress signalling and production of osmoprotectants.

Two of the strongest candidate genes for dehydration avoidance, *NAC4* and *SDIR* homologues, are likely to be involved in stress signalling pathways. NAC4 (nascent polypeptide-associated complex) is a transcription factor that is strongly upregulated in response to drought, salt and water stress in many species (Xia *et al.*, 2010; Shao *et al.*, 2015; Yu *et al.*, 2016) and confers drought tolerance when transgenically expressed in *Arabidopsis* (Mei *et al.*, 2021). More generally, the NAC gene family is involved in stomatal closure and lateral root development in rice (Hu *et al.*, 2006; Zheng *et al.*, 2009). SDIR1 (Salt and Drought-Induced Ring Finger1) is a RING-finger E3 ligase that is upregulated in *Arabidopsis* in response to drought stress and modulates both the ABA response and stomatal closure (Zhang *et al.*, 2007). Although upregulation of both genes in response to drought stress in our system is consistent with findings in other species and expected for dehydration avoidant plants, *sdir1* mutants in *Arabidopsis* had greater mortality following wilting than wild-type plants (Zhang *et al.*, 2007). This suggests an additional dehydration tolerance role for SDIR1 that we do not observe.

Expression of multiple genes that promote the synthesis of osmoprotectants [i.e. branched chain amino acids (BCAAs), proline and raffinose family oligosaccharides] are also strongly associated with lower wilt VWC. *BCAT2* (BCAA transaminase 2) catalyses the final transamination step in production of BCAAs, in addition to the first degradation step. Under stress, BCAAs accumulate as osmoprotectants in an ABA-dependent manner, with BCAT serving as the main regulator in *Arabidopsis*. P5CS, delta 1-pyrroline-5-carboxylate synthase, controls the rate-limiting step of proline biosynthesis from glutamate during osmotic and salt stress (Strizhov *et al.*, 1997). Transgenic overexpression of P5CS consistently increases tolerance to drought and salt stress across several plant species by increasing proline content (Su and Wu, 2004; Vendruscolo *et al.*, 2007; Yamchi *et al.*, 2007). *GOLS2* (galactinol synthase 2) encodes galactinol synthase 2, a key enzyme that regulates the first step in biosynthesis of raffinose family oligosaccharides, resulting in the creation of galactinol (Panikulangara *et al.*, 2004). Although the oligosaccharides act as osmoprotectants and as antioxidants, both galactinol and the oligosaccharides may also act as signalling molecules (Song *et al.*, 2016). In other species, GOLS activity is triggered by abiotic and biotic stress (*Arabidopsis*, coffee and rice) and confers drought tolerance (Dos Santos and Vieira, 2020). Unexpectedly, our results indicate that expression of *GOLS2* is linked with lower wilt VWC (greater dehydration avoidance) and with lower survival after wilting (lower dehydration tolerance). Increased expression of *GOLS2* in *Arabidopsis* does reduce transpiration rate (Taji *et al.*, 2002).

We also note a third process that might enhance dehydration avoidance and tolerance responses: the recycling and efficient use of nitrogen. *AlaAT2* (alanine aminotransferase 2) is strongly upregulated in plants that wilt at low VWC. We propose that this upregulation links drought response to nitrogen metabolism. Specifically, AlaAT2 catalyses the reversible conversion of alanine and α-oxoglutarate to pyruvate and glutamate, facilitating the synthesis of other amino acids as needed (Miyashita *et al.*, 2007). *AlaAT* homologues are upregulated during hypoxia in several species, including *Arabidopsis*, *Medicago*, poplar and corn, and are regulated by ABA in other stressful contexts (Ricoult *et al.*, 2005; Miyashita *et al.*, 2007; Xu *et al.*, 2017). Drought stress typically decreases the activity of nitrate reductase and nitrile reductase (Foyer *et al.*, 1998), impairing the ability of the plant to accumulate amino acids, owing to limited nitrogen assimilation or resources. Nitrogen fixation through symbiotic *Rhizobia* bacteria is also frequently impaired during drought stress (Zahran, 1999). Thus, even in legumes, useable nitrogen can be limited during periods of drought. Greater AlaAT2 activity could convert alanine into glutamate, which might then be used to produce BCAAs or proline as osmoprotectants. In sum, we hypothesize that the production of osmoprotectants requires carbon and nitrogen resources that might be procured most easily through recycling of existing metabolites.

The candidate genes associated with wilt survival generally do not have strong functional ties to drought responses in other systems, with two exceptions. *DegP5* (degradation of periplasmic proteins 5) belongs to a family of proteases that is typically upregulated in response to abiotic stress. Overexpression of *DegP5* has been linked to increased dehydration tolerance in *Nicotiana tabacum* via the suppression of Ca^2+^ and flagellin signalling, leading to a reduced accumulation of ROS (Chen *et al.*, 2023). In our study, increased expression of *DegP5* is also associated with wilting at higher soil moistures. This suggests that increased wilt survival comes at a cost of a less active drought response. The second exception is *SAPK2*, a plant-specific protein kinase in the family of SNF1-related protein kinase 2 (*SnRK2*) genes. Expression of the *SAPK2* homologue is positively associated with wilt survival (Fig. 5C). This role is similar to that documented in rice, where SAPK2 not only upregulates genes involved in osmoprotection but also induces genes involved in enzymatic ROS detoxification (Lou *et al.*, 2017). However, a role in dehydration avoidance has not been documented. Other SnRK2s do regulate cation channels (KAT1) and impact stomatal closure in *Arabidopsis* (Sato *et al.*, 2009). Other genes strongly associated with high wilt survival in our study function in other organisms in the detoxification of photorespiration byproducts (*PGLP2*; Schwarte and Bauwe, 2007) and in efficient light harvesting and photosynthesis (*LHCA6* and *CAB7*; Otani *et al.*, 2017). Together, these results suggest that improved survival after wilting is achieved through a combination of dampening responses to drought, protecting cells from harmful bioproducts of drought stress and maintaining metabolic functions.

### A trade-off between drought avoidance and tolerance strategies?

Drought resistance strategies are often presented as non-mutually exclusive, with the recognition that they represent different physiological mechanisms for a plant to acclimatize to low water availability. The relationship between drought escape and dehydration avoidance has been the most extensively scrutinized, because there are clear physiological trade-offs for C3 plants; closing stomata for better dehydration avoidance leads to lower carbon uptake and the inability to synthesize more sugars for rapid growth and reproduction (Geber and Dawson, 1990; Dudley, 1996). Theoretical and empirical identification of trade-offs between dehydration avoidance and tolerance is rare, potentially because there is not a true physiological trade-off that exists or because these strategies might be expressed sequentially as water stress increases (Volaire, 2018). We document relatively strong trade-offs between wilt VWC and wilt survival (Fig. 2), and expression responses for wilt VWC and wilt survival are strongly correlated (Fig. 4). However, the functions of genes associated with each of these phenotypes suggest that our experiment is not truly measuring separate dehydration avoidance and tolerance responses. Rather, our wilt VWC treatment is likely to be catching both dehydration avoidance and tolerance responses; plants that survive to lower wilting are effectively tolerating dehydration for longer than plants that wilted earlier, probably by increasing osmoprotectants and by closing stomata to regulate gas exchange. Correlated dehydration avoidance and tolerance responses make sense, because both functions are regulated, at least in part, by ABA. However, given that dehydration tolerance responses often involve the production of various osmoprotectants and closing stomata (which limits CO_2_ and useable plant nitrogen uptake), correlated physiological responses must require recycling of carbon and nitrogen.

The strong correlation between wilt VWC and wilt survival is more likely to represent a trade-off between different elements of dehydration tolerance. Wilting occurs when the turgor pressure within cells falls to zero, leading to potential cellular damage. This damage is exacerbated under prolonged drought stress, because increased ROS production can overwhelm the antioxidant defences of the plant and, coupled with stomatal closure, can also increase photorespiration flux (Noctor *et al.*, 2002). To survive wilting, plants must be able to protect cellular function and integrity. Our study found that plants with better wilting survival had higher expression of genes associated with antioxidant defence (*DegP5*) and detoxification of photorespiration byproducts (*PGLP2*). Although expression of each of these genes was associated with high wilt VWC, the only truly strong correlation was for *DegP5*, a gene linked to diminishing Ca^2+^ signalling and lower ROS production in tobacco (Chen *et al.*, 2023). Expression of this gene might represent a true trade-off between different physiological strategies. Generally, our results suggest that a dehydration tolerance strategy represents multiple different types of tolerance: one strategy that provides resistance against dehydration and another that allows longer-term survival following wilting (i.e. extreme dehydration). We hypothesize that dehydration tolerance related to drought survival might have overlapping physiology with desiccation tolerance strategies occurring in plants such as the resurrection fern. In sum, connecting variation between physiological processes and plant performance will provide additional insight into the evolution of different ecological strategies in specific environments.

### Loci underlying drought response adaptation following introduction

Identifying the causative molecular variants that underlie natural intraspecific variation in drought responses has proved challenging, with limited successes (Juenger, 2013; VanWallendael *et al.*, 2019). This objective is largely beyond the scope of our study. We do identify many genes that are both linked with wilt VWC or wilt survival phenotypes and show variable expression across ranges, conditions and latitudes. This overlap includes a substantial portion of the turquoise gene co-expression network, suggesting that variation in dehydration avoidance and tolerance phenotypes is manifested through broad shifts across gene expression networks (e.g. Lovell *et al.*, 2018) rather than through simple, large-effect variants that directly cause lower stomatal conductance or greater osmoprotection (e.g. Kesari *et al.*, 2012; Des Marais *et al.*, 2014).

Recent work has identified several haploblocks that underlie climate adaptation following introduction to North America, in addition to several candidate genes within haploblocks that were differentially expressed across drought treatment, range and latitude (Battlay *et al.*, 2024). Within the three haploblocks on Chr04_Occ (synonym Chromosome 7), 26 differentially expressed transcripts were found (9 on HB7a1; 14 on HB7a2; and 3 HB7b; Supplementary Data Table S9). On HB7a1, four genes within the turquoise submodule were differentially expressed. Two turquoise submodule genes, *P5CS1* and *FER1*, were strongly correlated with wilt VWC and wilt survival. In addition, *BRR2A* and *DWF5* were correlated significantly with wilt survival, whereas *IAA6* and *DWF5* were correlated significantly with wilt VWC. On HB7b, transcripts belonging to the gene *CCL12* are significantly correlated with wilting. Several of these genes have allelic variation associated with higher fitness in North American common gardens (Albano *et al.*, 2024; Battlay *et al.*, 2024). These results provide additional evidence for the importance of regulatory variation in rapid adaptation during invasion.

### Conclusions

Our work suggests that selection acts on rapid time scales following introduction to allow more precise adaptation of drought responses to divergent environmental conditions, with trade-offs occurring between drought resistance strategies. We identify a co-expression network that underlies both dehydration avoidance and some elements of dehydration tolerance, whereby many of these loci also exhibit differential expression patterns between the native and introduced ranges of white clover. We also identify trade-offs at the molecular level among functions (osmoprotection and detoxification) typically identified within the dehydration tolerance strategy; a result that needs to be considered carefully in breeding efforts. Our results are the first step to tying locally adaptive genetic variation to variation in gene regulatory networks, physiology and ecological strategies. We hope this work stimulates more studies examining and relating genetic variation and expression networks to phenotypic differences within natural populations to create a broader consensus on mechanisms creating variation in drought resistance and survival.

## SUPPLEMENTARY DATA

Supplementary data are available at *Annals of Botany* online and consist of the following.

Table S1: geographical and environmental characteristics of white clover populations. Table S2: mapping statistics from RNAseq experiment. Table S3: univariate model results examining associations between drought strategy phenotypes across ranges, latitudes and treatments. Table S4: univariate model results examining associations between drought strategy phenotypes with water availability-related variables of collection populations. Table S5: summary of differentially expressed genes with associated modules and phenotypic associations. Table S6: Gene Ontology analysis of WGCNA modules. Table S7: genes with variation in expression associated with wilt volumetric water content or wilt survival. Table S8: genes with significant range–phenotype interactions. Table S9: patterns of expression within haploblocks. Figure S1: volumetric water content of pots within well-watered and dry-down treatments throughout the experiment. Figure S2: relationships between drought strategy phenotypes and number of leaves at the start of the experiment. Figure S3: associations between water availability-related variables of collection populations and number of leaves at the start of the experiment. Figure S4: associations between water availability-related variables of source populations and drought avoidance and tolerance. Figure S5: Venn diagram of differentially expressed genes across latitude, range and treatment contrasts. Figure S6: normalization expression profiles across samples. Figure S7: relative expression across treatment, region and latitude for candidate genes.

mcaf037_suppl_Supplementary_Tables_S1-S9

mcaf037_suppl_Supplementary_Figures_S1-S7

## Data Availability

Phenotypic data from the growth chamber experiment is available via Dryad: https://doi.org/10.5061/dryad.hqbzkh1tt. RNAseq data is available via NCBI’s short read archive: Bioproject PRJNA1098360.
